# Virulence Characteristics and Antimicrobial Resistance Profiles of Shiga Toxin-Producing *Escherichia* *coli* Isolates from Humans in South Africa: 2006–2013

**DOI:** 10.3390/toxins11070424

**Published:** 2019-07-19

**Authors:** Musafiri Karama, Beniamino T. Cenci-Goga, Mogaugedi Malahlela, Anthony M. Smith, Karen H. Keddy, Saeed El-Ashram, Lawan M. Kabiru, Alan Kalake

**Affiliations:** 1 Department of Paraclinical Sciences, Faculty of Veterinary Science, University of Pretoria, Onderstepoort 0110, South Africa; 2 Dipartimento di Medicina Veterinaria, Laboratorio di Ispezione degli Alimenti di Origine Animale, University of Perugia, 06126 Perugia, Italy; 3 Centre for Enteric Diseases, National Institute for Communicable Diseases, Johannesburg 2131, South Africa; 4 College of Life Science and Engineering, University Foshan, Foshan 528231, China; 5 Faculty of Science, Kafrelsheikh University, Kafrelsheikh 33516, Egypt; 6 Department of Veterinary Public Health and Preventive Medicine, Ahmadu Bello University, Zaria 2222, Nigeria; 7 Gauteng Department of Agriculture and Rural Development, Johannesburg 2001, South Africa

**Keywords:** STEC, human, virulence, antimicrobial resistance, PFGE

## Abstract

Shiga toxin-producing *Escherichia coli* (STEC) isolates (N = 38) that were incriminated in human disease from 2006 to 2013 in South Africa were characterized by serotype, virulence-associated genes, antimicrobial resistance and pulsed-field gel electrophoresis (PFGE). The isolates belonged to 11 O:H serotypes. STEC O26:H11 (24%) was the most frequent serotype associated with human disease, followed by O111:H8 (16%), O157:H7 (13%) and O117:H7 (13%). The majority of isolates were positive for key virulence-associated genes including *stx1* (84%), *eaeA* (61%), *ehxA* (68.4%) and *espP* (55%), but lacked *stx2* (29%), *katP* (42%), *etpD* (16%), *saa* (16%) and *subA* (3%). *stx2* positive isolates carried *stx2c* (26%) and/or *stx2d* (26%) subtypes. All pathogenicity island encoded virulence marker genes were detected in all (100%) isolates except *nleA* (47%), *nleC* (84%) and *nleD* (76%). Multidrug resistance was observed in 89% of isolates. PFGE revealed 34 profiles with eight distinct clusters that shared ≥80% intra-serotype similarity, regardless of the year of isolation. In conclusion, STEC isolates that were implicated in human disease between 2006 and 2013 in South Africa were mainly non-O157 strains which possessed virulence genes and markers commonly associated with STEC strains that have been incriminated in mild to severe human disease worldwide. Improved STEC monitoring and surveillance programs are needed in South Africa to control and prevent STEC disease in humans.

## 1. Introduction

Shiga toxin-producing *Escherichia coli* (STEC) is a zoonotic foodborne and waterborne pathogen associated with enteric disease in humans characterized by abdominal cramps, mild to severe diarrhea, hemorrhagic colitis (HC) and hemolytic uremic syndrome (HUS) [1]. Majowicz et al. [2] have estimated that STEC is responsible for 2.8 million cases per year of enteric disease in humans, globally. Humans acquire STEC through ingestion of contaminated foods of animal origin, water and raw vegetables or contact with infected animals [3,4,5].

More than 600 STEC serotypes have been recovered from animals, humans, the environment and various foods around the world [6,7,8]. STEC O157:H7 is the most common serotype in human infections, but non-O157 STEC have also emerged as an important cause of enteric disease [6,7,8,9] and may account for up to 80% of human STEC infections [2,10]. Furthermore, most STEC infections in humans have been ascribed to STEC strains belonging to seven major serogroups, including STEC O157, O26, O111, O103, O145, O121 and O45 [7,11].

Shiga toxins (*stx1* and *stx2*) are the main virulence factors of STEC. At least 15 Shiga toxin subtypes have been identified (http://old.iss.it/binary/vtec/cont/STEC_2018_Wrap_up.pdf), including *stx1a*, *stx1c*, *stx1d*, *stx1e*, *stx2c*, *stx2d*, *stx2e*, *stx2f*, *stx2g*, *stx2h*, *stx2i*, *stx2j*, *stx2k* and *stxl* [12,13,14]. Another key virulence factor of STEC is the *E. coli* attachment and effacement gene (*eae*) which encodes intimin (*eaeA*) [15,16,17]. Intimin (*eaeA)* is encoded on the locus of the enterocyte effacement (LEE) pathogenicity island [16] and is responsible for attaching and effacing (AE) lesions which are observed in the intestinal epithelium of human and animal hosts affected with *eaeA* positive *E. coli*, including STEC [16,17].

STEC produce plasmid-encoded virulence-associated proteins, including hemolysin (*hlyA* or *ehxA*) [18], catalase peroxidase (*katP*) [19], extracellular serine protease (*espP*) [20] and a type II secretion pathway (*etpD*) [21]. STEC autoagglutinating adhesin (*saa)* and subtilase cytotoxin (*subA*) are also recognized as key plasmid-encoded virulence factors in *eaeA* negative STEC strains [22,23]. Plasmid-encoded virulence genes have been associated with increased adherence and colonization (*saa*) of enterocytes, cytotoxicity (*subA*), reduction of oxidative stress (*katP*), exoprotein secretion (*etpD*), suppression of the host’s immune system and cleavage of coagulation factor V (*espP*) and lysis of human erythrocytes with subsequent mucosal hemorrhage in the host (*hlyA*) [18,20,21,22,23,24,25].

Several genes that are encoded on pathogenicity islands (O-islands-OIs) other than the LEE are also considered STEC virulence markers [26]. Particularly, genes located on OI-122 [11,27], including *pagC*, which encodes a protein sharing 46% homology with the *phoP*-activated gene C product in *Salmonella enterica* serovar Typhimurium. PagC enables survival in macrophages. Another OI-122–encoded gene is *sen*, which encodes a protein that is 38.2% homologous to *Shigella flexneri* enterotoxin 2 [11,27], as well as *efa1* and *efa2* (enterohemorrhagic *E. coli* factor for adherence), which have been associated with epithelial cell adhesion and inhibition of bovine peripheral blood lymphocyte proliferation [11,28]. OI-122 marker genes have been used to characterize and classify STEC serotypes into seropathotypes based on their association with outbreaks and severe disease complications, such as HC and HUS in humans [11]. In addition, genes that are carried on OI-43/48 are also considered key virulence markers: *iha* (iron-regulated gene A), which encodes an adhesin [29] and *ureC* [30,31] and *terC* [31], which encode urease and tellurite resistance, respectively. Furthermore, numerous non-LEE encoded effector (nles) genes that are carried on various pathogenicity islands are considered virulence markers: *nleA* (OI-71), *nleB*/*Z4328* (OI-122), *nleB2*, *nleC* (OI-36), *nleD* (OI-57), *nleE*/*Z4329* (OI-122), *nleG* (OI-71), *nleG2-1* (OI-71), *nleG2-3* (OI-57), *nleG6-2* (OI-57), *nleG9* (OI-71), *nleH1-2* (OI-171) [32] and *ent/espL2* (OI-122). These genes are mainly found in high risk (HUS) STEC strains and are associated with colonization and survival in the host, and may interfere with signaling pathways during inflammation [33]. Non-LEE encoded effector genes have been used in molecular epidemiology to differentiate highly virulent from less virulent STEC strains (molecular risk assessment) [32,34,35].

The emergence of antimicrobial resistant bacteria as a consequence of the indiscriminate use and abuse of antimicrobials in animals and humans has become a public health concern [36,37,38,39]. Currently, the use of antimicrobials for treatment of STEC infections in humans is controversial and not recommended in many countries, because a number of studies have shown that antimicrobials, such as ciprofloxacin and trimethoprim-sulphamethoxazole, can induce STEC lysogenic bacteriophages to enter the lytic cycle, which exacerbates STEC infections by increasing Shiga toxin production with resultant severe STEC disease manifestations in humans [39,40]. In addition, STEC bacteriophages may carry genes encoding antimicrobial resistance which can be transferred to naïve *E. coli*, which are then converted into antimicrobial resistant strains. Therefore, *E. coli*, including STEC, as commensal bacteria in the intestinal tracts of animals and humans are considered reliable indicators of antimicrobial resistance, and have been used for monitoring and surveillance of antimicrobial resistance in livestock, humans and along the food chain.

Although the first case and largest outbreak of human STEC disease in South Africa occurred in 1990 and 1992, respectively [41,42], ten years after the first report of a human STEC disease outbreak in the world in the USA [43], data on the epidemiology, virulence characteristics, antimicrobial resistance patterns and genotypes of human STEC isolates from South Africa remain scanty. In this study, STEC isolates that were incriminated in human disease outbreaks in South Africa from 2006–2013 were fully serotyped, screened for virulence-associated genes and antimicrobial resistance patterns and subtyped by pulsed-field gel electrophoresis (PFGE). The overall aim of this study was to contribute to STEC surveillance and gain insight into the molecular epidemiology of STEC isolates that have been implicated in human disease in South Africa.

## 2. Results

### 2.1. STEC Serotypes and Their Frequency in Human Outbreaks

Serotyping (O:H) assigned the 38 STEC isolates to 11 O:H serotypes, including 11 O serogroups and 6 H types. The isolates belonged to the following serotypes: O5:HNT (2), O8:H19 (1), O22:H16 (1), O26:H11 (9), O107:H7 (3), O111:H8 (6), O113:H21 (1), O117:H7 (5), O118:H16 (1), O156:H7 (2), O157:H7 (5), ONT:H8 (1) and ONT:H21 (1) (Table 1). Overall, STEC O157:H7 accounted for 13.1% (5/38) and non-O157 serotypes were associated with 87% (33/38) of STEC isolates that caused human disease in South Africa from 2006 to 2013. STEC O26:H11 was the most frequent serotype, responsible for 24% (9/38) of human disease cases which occurred over five years (2007, 2008, 2009, 2010 and 2013), followed by STEC O111:H8, which was incriminated in 16% (6/38) of cases which occurred over four years (2006, 2008, 2009 and 2010). STEC O157:H7 and O117:H7 were associated with 13% (5/38) of human disease cases for each individual serotype, which occurred over four (2009, 2010, 2011 and 2012) and three years (2006, 2010, and 2012), respectively, while STEC O107:H7 was implicated in 8% (3/38) of cases which all occurred in 2007. The remaining serotypes (O5:HNT, O8:H19, O22:H16, ONT:H8, O118:H16, ONT:H21 and O156:H7) were implicated once or twice in human disease in the period spanning from 2006–2013.

### 2.2. Virulence-Associated Gene Distribution

**Figure 1:**
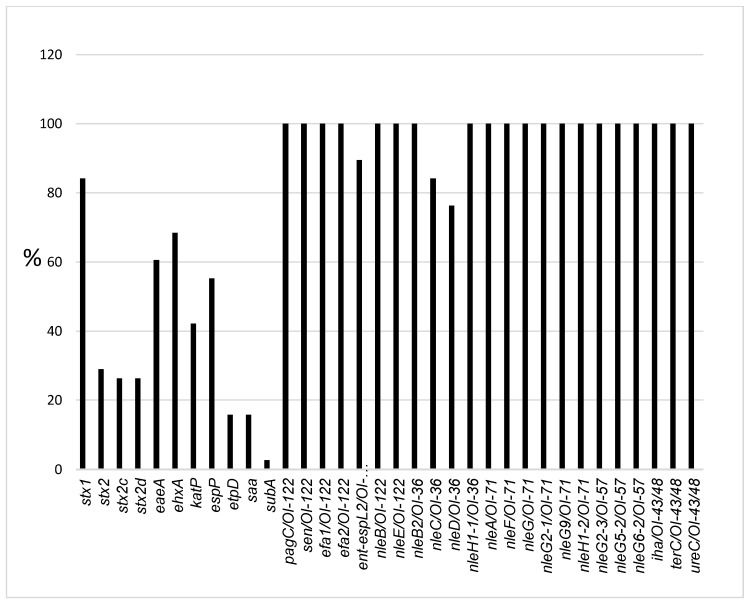
Distribution of virulence-associated genes in STEC isolates from humans in South Africa.

The frequencies of virulence genes are depicted in Figure 1. PCR revealed that overall, 84% (32/38) of isolates carried *stx1*, 29% (11/38) carried *stx2*, 26% (10/38) carried *stx2c* and 26% (10/38) carried *stx2d* (Table 1 and Figure 1). Both *stx1* and *stx2* were detected concurrently in 13% (5/38) of isolates while both *stx1* and *stx2c* were detected in 0.5% (2/38) of isolates. *stx1* alone or *stx2* alone were detected in 66% (25/38) and 16% (6/38) of isolates, respectively. The following *stx* subtypes were not detected: *stx1c* and *stx1d*, *stx2e*, *stx2f* and *stx2g*. Additional genotypes were also observed: *stx1* + *stx2c* in 8% (3/38), *stx1* + *stx2* + *stx2d* in 8% (3/38), *stx2* + *stx2c* + *stx2d* in 13% (5/38) and *stx1 + stx2 + stx2c + stx2d* in 5% (2/38) of isolates. Both *stx2c + stx2d* were detected concurrently in 18% (7/38) of isolates which belonged to the following serotypes: STEC O157:H7 (5), STEC O8:H19 (1) and STEC O113:H2 (1). The *eaeA* gene was detected in 60.5% (23/38) of isolates which belonged to the following serotypes: STEC O5:HNT, O26:H11, O111:H8, O118:H16, O157:H7 and ONT:H8. 

Plasmid-encoded genes were distributed as follows: *ehxA*, 68.4% (21/38); *katP*, 42% (16/38); *espP,* 55% (21/38); *etpD*, 16% (6/38) and *saa,* 16% (6/38) (Table 1 and Figure 1). Only one isolate carried *subA*. A complete plasmid (*ehxA*, *katP*, *espP* and *etpD*) was detected in STEC O157:H7 isolates only while O26:H11 isolates possessed all plasmid marker genes except *etpD*. The following OI marker genes were present in 100% of isolates: OI-122 markers: *pagC*, *sen*, *efa1* and *efa2* except *ent*/*espL2,* which was detected in 89.4% of isolates; OI-43/48 markers: *terC*, *ureC* and *iha* were present in 100% of isolates. Most *nle* effector genes, including *nleB*, *nleB2, nleF*, *nleE*, *nleG*, *nleG2-1*, *nleG2-3*, *nleG5-2*, *nleG6-2*, *nleG9*, *nleH1-1* and *nleH1-2*, were present in 100% of isolates, while *nleA* was found in 47% (18/38), *nleC* in 84% (32/38), and *nleD* in 76% (29/38).

Overall, the STEC isolates carried 50–74% (20–28 out of 34) genes. STEC O157:H7 carried 28/34 genes, STEC O118:H16 had 27/34 genes, STEC O26:H11 had 26–27/34 genes, STEC O5:HNT had 25–26/34 genes, STEC O8:H19 had 25/34 genes and two STEC O111:H8 isolates had 25/34 genes. The remaining serotypes, including STEC O111:H8, O113:H21, O107:H7, O117:H7, O22:H16, O156:H7, ONT:H21 and ONT:H8, carried 21–24/34 genes. 

### 2.3. Antimicrobial Resistance Profiling

**Figure 2:**
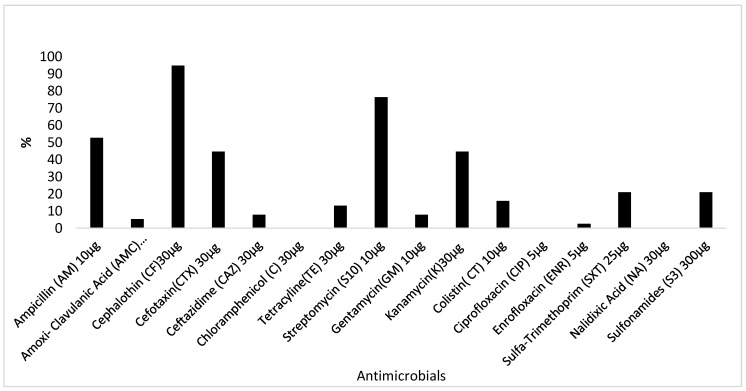
Antimicrobial resistance profiles of STEC isolates from humans in South Africa.

The distribution of antimicrobial resistance patterns is depicted in Figure 2. All the 38 STEC isolates were resistant to one or more antimicrobial. The following resistance rates were recorded per antimicrobial: cephalotin, 95% (36/38); streptomycin, 76% (29/38); ampicillin, 53% (20/38); amoxicillin-clavulanic acid, 5% (2/38); cefotaxin and kanamycin, 45% (17/38); sulfonamides and sulfa-trimethoprim, 21% (8/38); colistin, 16% (6/38); tetracycline, 13% (5/38) and ceftazidime and gentamycin, 8% (3/38). None of the isolates were resistant to chloramphenicol, ciprofloxacin, enrofloxacin or nalidixic acid. Multi-resistance (resistance to two or more classes of antimicrobials) was observed in 89% (34/38) of isolates. Two main multi-resistance patterns were detected: ampicillin/cephalothin/streptomycin, 42% (16/38) and cephalothin/streptomycin, 32% (12/38). Resistance to five or more antimicrobials was detected in 29% (11/38) of isolates.

### 2.4. Pulsed-Field Gel Electrophoresis

**Figure 3:**
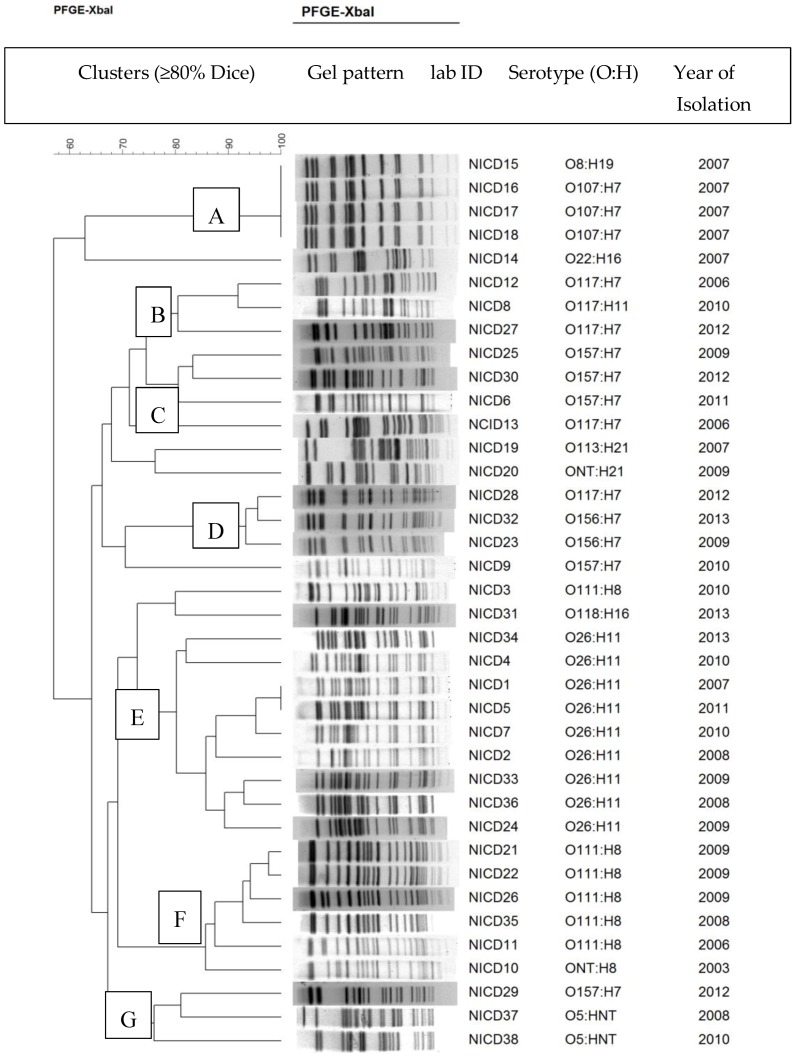
Dendrogram showing relationships among STEC isolates that were incriminated in human disease in South Africa from 2006 to 2013.

PFGE was performed to investigate genetic relationships among isolates. The 38 STEC isolates were all typeable by PFGE. The isolates were classified into 33 PFGE profiles. Based on a Dice similarity index ≥80%, the 33 PFGE profiles were grouped into seven distinct clusters (A, B, C, D, E, F, and G) comprising mainly three to nine isolates/per cluster (Figure 3). STEC that belonged to the same serotype clustered together regardless of the year of isolation. Two major clusters emerged: cluster E, which grouped all the nine STEC O26:H11 isolates which were implicated in human disease in 2007, 2008, 2009, 2010, 2011 and 2013 and cluster F, which included five STEC O111:H8 isolates which were incriminated in human infections in 2006, 2008 and 2009. The remaining five clusters (A, B, C, D and G) were minor clusters of less than five isolates per cluster.

## 3. Discussion

Serotyping, virulence characterization, antimicrobial resistance and PFGE genotyping of STEC isolates that are recovered from infected humans are used in high income countries with robust STEC monitoring and surveillance programs for risk assessment purposes. While current data on the molecular epidemiology of STEC are readily available in high income countries, information on the occurrence and characteristics of STEC isolates from infected humans in low income countries, such as South Africa and most middle income economies, is scarce. Currently, STEC surveillance in South Africa remains passive and STEC disease in South Africa may be underestimated due to lack of active surveillance and/or underreporting. In addition, with cattle being an important STEC reservoir [44], the apparent sporadic nature of the disease in humans may be an inaccurate reflection of the true prevalence of STEC infection in humans. In this study, STEC isolates that were implicated in sporadic human disease in South Africa between 2006 and 2013 were characterized for virulence-associated genes and antimicrobial resistance profiles. Isolates were also genotyped by PFGE. STEC O26:H11 was the most frequent serotype associated with human disease, followed by O111:H8 and O157:H7 and O117:H7, contrary to world trends which have implicated STEC O157:H7 as the most frequent serotype associated with human infections [2]. Furthermore, the STEC isolates which were implicated in human disease in South Africa from 2006–2013 were mostly non-O157 (86.8%) serotypes, in agreement with global trends which have shown that non-O157 STEC are responsible for the majority of human disease cases worldwide [2,9]. 

Overall, the STEC isolates belonged to 11 O:H serotypes including O5:HNT, O8:H19, O22:H16, O26:H11, O107:H7, O111:H8, O113:H21, O117:H7, O118:H16, O156:H7, O157:H7, ONT:H8 and ONT:H21. Apart from STEC O107:H7, all the isolates belonged to serotypes which have been previously incriminated in mild to severe human STEC disease in various countries worldwide [6,7,8,9,45]. Altogether, three “big 7” STEC serotypes, including STEC O26:H11, O111:H8 and O157:H7, accounted for 53% of all isolates that were associated with human disease in South Africa from 2006–2013. This is consistent with previous reports which have shown that STEC O26:H11, O111:H8 (16%) and O157:H7 are among the three most frequent serotypes that are commonly associated with human disease globally [6,7,8,9,45]. 

The vast majority of STEC isolates were *stx1* positive (84%), while only a low number (29%) possessed *stx2.* No *stx1* subtypes were detected in this study. The *stx1 + eaeA* genotype was mostly observed among STEC O26:H11 and O111:H8 strains. STEC that carry *stx1* alone or both *stx1* + *eaeA* are considered low risk and are commonly associated with mild diarrhea or non-HUS infections in humans [46,47]. However, in some cases, *stx1 + eaeA* positive STEC have been involved in severe disease and outbreaks, suggesting that some *stx1* + *eaeA* positive STEC strains may have yet unidentified virulence factors that make them highly virulent [48]. 

Almost all *stx2* positive isolates carried both *stx2c* and *stx2d* subtypes concurrently. STEC isolates that possessed *stx2* exhibited *stx2 + stx2c + stx2d + eaeA* or *stx2 + stx2c + stx2d* gene combinations. *stx1 + stx2 + stx2c + stx2d + eaeA* or *stx2 + stx2c + stx2d + eaeA* were observed in STEC O157:H7 isolates only while the *stx2 + stx2c + stx2d* genotype was detected in STEC O113:H21. The *stx1 + stx2 + stx2d* genotype was detected in STEC O107:H7 isolates, while the *stx1 + stx2 + stxc + stx2d* genotype was observed in one STEC O8:H19 isolate. STEC that possess *stx2* alone or *stx2 + eaeA* have been significantly associated with severe disease in humans, including HC and HUS, in comparison to STEC that possess *stx1* alone or *stx1 + stx2* concurrently [46,47]. The proportion of individual STEC isolates that carried both *stx2c* and *stx2d* concurrently was higher compared to the results of two studies, which previously reported single strains that possess both subtypes [12,49]. The presence of both *stx2c* and/or *stx2d* subtypes in a STEC isolate has been associated with human disease, varying from mild to bloody diarrhea and HUS [12,49,50,51,52].

The *eaeA* gene was detected in the majority of isolates (60.5%). The *eaeA* gene was observed in the following serotypes: STEC O5:HNT, O26:H11, O111:H8, O118:H16, O157:H7 and ONT:H8. The high frequency of *eaeA* in these human STEC isolates is in sharp contrast with the very low rates of *eaeA* found in STEC isolates from cattle in South Africa [44], which are considered an important STEC reservoir, suggesting that only a small subset of cattle STEC that are *eaeA* positive with a high capacity of being easily transmitted to humans may be involved in human disease in South Africa.

The majority of STEC isolates carried plasmid-encoded virulence markers *ehxA*, *katP* and *espP* but lacked *etpD, saa* and *subA.* A complete plasmid (pO157) (*ehxA*, *katP*, *espP* and *etpD*) was observed in O157:H7 isolates only, while O26:H11 isolates possessed *ehxA*, *katP*, *espP* and *etpD* but lacked *etpD,* which was only found in all STEC O157:H7 and O5:HNT strains. The plasmid-encoded genes *katP*, *espP* and *etpD* are more frequent in STEC isolates that are recovered from human disease outbreaks and cases of HUS, particularly [11,28]. As previously shown, the *saa* gene was present in only a small number of *eaeA*-negative STEC isolates [53,54] which belonged to serotypes O113:H21, O8:19 and O107:H7. STEC O113:H21 has been previously incriminated in cases of severe human STEC infections (HUS) in Australia and Europe, while STEC O8:H19 has been associated with diarrhea in various countries [7,23]. To the best of our knowledge, this is the first report that has incriminated STEC O107:H7 in human disease, suggesting that STEC O107:H7 may be an emerging serotype worth monitoring in South Africa.

Most of the isolates carried the full complement of OI-122 and OI-43/48 marker genes and all non-LEE encoded effector genes, except *nleA*. OI-122 and OI-43/48 and non-LEE effectors genes encode proteins that play an important role in STEC virulence as substrates that are translocated through a type III secretion system. Overall, significantly more OI-122, OI-43/48 and non-LEE effector genes were observed in STEC isolates that were *stx2* and *eaeA* positive with a complete plasmid (*ehxA*, *katP*, *espP* and *etpD*), in agreement with Ju et al. [55]. Possession of OI-122, OI-43/48 and the non-LEE effector genes together with *stx2*, *eaeA* and a complete plasmid is a hallmark of highly virulent STEC strains that are frequently associated with outbreaks and severe disease such as HC and HUS [11,56,57], whereas serotypes lacking these genes are mostly implicated in mild human disease [11,32,57,58].

Interestingly, STEC O157:H7, O26:H11, O111:H8, O113:H21, O118:H16, O5:HNT and O8:H19 harbored the highest number of virulence genes (24–28 genes). Possession of the highest number of virulence-associated genes significantly correlated with the presence of *eaeA*. While the presence of a high number of virulence genes in STEC O157:H7, O26:H11, O111:H8 and O113:H21 isolates that are commonly implicated in severe foodborne STEC disease outbreaks around the world may not be surprising [6,7,8,9,11], possession of a high number of virulence-associated genes in STEC O118:H16, O5:HNT and O8:H19 serotypes that are rarely associated with severe disease and outbreaks globally [6,7] suggests that these serotypes should be closely monitored in South Africa as they may present a high risk for humans.

All of the 38 STEC isolates were resistant to one or more antimicrobials. Most (>50%) of the isolates were resistant to cephalothin, streptomycin and ampicillin, while moderate resistance rates (40–50% of isolates) were observed for cefotaxin and kanamycin, and lower resistance levels (< 25%) were observed for sulfonamides, sulfa-trimethoprim and colistin. The levels of resistance recorded in this study were very high compared to rates that have been reported elsewhere [59,60,61]. Isolates were multi-resistant to ampicillin/cephalothin/streptomycin, or cephalothin/streptomycin, mainly. These antimicrobials are commonly used for the treatment of human bacterial diseases in clinical medicine. The high levels of resistance against these antimicrobials suggests that these compounds may be misused or abused in clinical medicine and are exerting selective pressure on the STEC isolates under study.

Pulsed-field gel electrophoresis was used to classify the 38 STEC isolates into 33 profiles which were assigned to eight distinct clusters. Among the eight clusters, four distinct clusters grouped STEC O26:H11, five of the six STEC O111:H8 and all STEC O107:H7 and O157:H7. The four clusters were serotype specific with very high intra-cluster similarity (>80%), regardless of the year of isolation. The high intra-serotype similarity among STEC O26:H11, O111:H8, O107:H7 and O156:H7 strains regardless of their year of isolation suggests that the STEC isolates which were implicated in disease in South Africa from 2006 to 2013 are clonally related STEC strains which may have a high capacity for survival and persistence in their respective reservoir or sources.

## 4. Conclusions

In summary, the majority of STEC infections which occurred in South Africa from 2006 and 2013 were caused by serotypes O26:H11, O111:H8 and O157:H7 that belong to “big 7” serogroups, in agreement with global trends. Virulotyping revealed that most of the isolates were *stx1* + *eaeA* positive, fewer isolates carried *stx2* only or concurrently with *stx2c* and *stx2d* and a smaller number was positive for both *stx1* and *stx2*. Most isolates carried all or most pathogenicity island encoded virulence-associated genes. PFGE revealed high intra-serotype similarity among STEC. The majority of isolates were resistant to two or more antimicrobials that are commonly used in clinical medicine for treatment of various bacterial diseases. To the best of our knowledge, this is the first detailed characterization of human STEC isolates from South Africa. Further molecular epidemiology studies that compare STEC isolates of human, animal and food origin are needed to fully understand the epidemiology of STEC and identify reservoirs and sources of human disease in South Africa.

## 5. Materials and Methods

### 5.1. STEC Strains and Culture Conditions

A total of 38 STEC isolates which were previously recovered from humans showing foodborne disease symptoms, including mild to severe diarrhea, between 2006 and 2013 were characterized in this study. These isolates were obtained from the culture collection of the Centre for Enteric Diseases, National Institute of Communicable Diseases, South Africa (CED-NICD). Because the isolates were obtained through passive surveillance, detailed clinical information on patients from which the STEC isolates were recovered was unavailable. The 38 STEC isolates belonged to the following serogroups: O5 (2), O8 (1), O107 (3), O22 (1), O26 (9), O111 (6), O113 (1), O117 (5), O118 (1), O156 (2), O157 (5) and ONT (1). This study was approved by the University of Pretoria Research Ethics Committee (REC005-18).

### 5.2. STEC Cultures 

The isolates were resuscitated on Luria Bertani Agar after incubation at 37 °C overnight. DNA was extracted from bacterial cells by the boiling method, as described previously [59]. Briefly, a sterile inoculating loop was used to harvest pure *E. coli* colony sweeps from Luria Bertani Agar plates. A loop-full of colony sweeps was suspended in a 1.5 mL Eppendorf tube containing 1 mL of FA Buffer (Becton Dickinson and Company, Sparks, MD, USA). Bacterial suspensions were mixed and washed by vortexing, followed by centrifugation (15,000× *g*) for 5 min. After the first wash and centrifugation cycle, the supernatant was discarded and the bacterial pellet was re-suspended in FA buffer (Becton, Dickinson and Company Sparks, MD, USA). After two additional washes and centrifugation cycles, the pellet was suspended in 500 µL of sterile water and thoroughly vortexed. The homogeneous cell suspension was boiled [62] to 100 °C for 15 min, then stored at −20 °C until further processing.

### 5.3. STEC PCR Serotyping (O:H)

STEC isolates were further serotyped for flagellar antigens (H antigens) only, as STEC serogrouping (O antigen typing) had been carried out previously by the tube agglutination test (antisera manufactured by the Statens Serum Institut, Copenhagen, Denmark). H typing was carried out by three multiplex PCR assays that targeted 14 genes encoding *Escherichia coli* flagellar genes (*fliC*) that are commonly found in STEC [63]. 

### 5.4. Virulotyping

Virulotyping was performed using previously described primers and PCR cycling conditions to amplify 34 genes that encode various STEC virulence factors and markers. A multiplex PCR was used for the detection of *stx1, stx2*, *eaeA*, and *ehxA* [64]. Detection of *stx2* subtypes (*stx1c, stx1d, stx2c, stx2d, stx2e, stx2f* and *stx2g*) was carried out according to Scheutz et al. [12]. Single PCR reactions were conducted to amplify plasmid-encoded virulence markers: *katP* [19], *espP* [20], *etpD* [21], *saa* [64] and *subA* [65]. Previous PCR protocols with slight modifications were used to screen for OI-encoded genes including OI-43/48: *iha*, *ureC* and *terC* [29,30,31] and OI-122: *pagC* (*Z4321*), *sen* (*Z4326*), *efa1* (*Z4332*) and *efa2* (*Z4333*) [11]. The PCR protocols of Coombes et al. [32] were used to search for non-LEE- encoded effector genes: *nleA* (OI-71), *nleB* (OI-122), *nleC* (OI-36), *nleD* (OI-57), *nleE* (OI-122), *nleG* (OI-71), *nleG2-1* (OI-71), *nleG2-3* (OI-57), *nleG6-2* (OI-57), *nleG9* (OI-71) and *nleH1-2* (OI-171). STEC O157:H7 strain EDL 933 (*E. coli* O157:H7) was used as a positive control. 

### 5.5. Antimicrobial Susceptibility Testing

Antimicrobial resistance profiles of STEC isolates were determined against a panel of 12 antimicrobials by the disk diffusion method as described by the Clinical and Laboratory Standards Institute (CLSI) [66]. The panel of antimicrobials consisted of ampicillin (10 µg), amoxicillin-clavulanic acid (20 and 10 µg, respectively), cephalothin (30 µg), cefotaxin (30 µg), ceftazid (30 µg), chloramphenicol (30 µg), tetracycline (30 µg), streptomycin (10 µg), gentamicin (10 µg), kanamycin (30 µg), colistin (10 µg), ciprofloxacin (5 µg), enrofloxacin (5 µg), sulfa-trimethoprim (1.25 and 23.75 µg, respectively), nalidixic acid, (30 µg) and sulfonamides (300 µg). Antimicrobial disks (BBL Sensi Disk) were obtained from Becton, Dickinson and Company, Sparks, MD, USA) and Oxoid, Basingstoke, Hampshire, England). *E. coli* ATCC 25922 was used as the control strain. Isolates were classified as susceptible, intermediate or resistant to each antimicrobial agent and in the final analysis intermediate readings were assigned to the resistant category. 

### 5.6. Pulsed-Field Gel Electrophoresis

PFGE was carried out according to the CDC/PulseNet protocol to determine relationships among STEC, particularly STEC belonging to the same serotype (http://www.cdc.gov/pulsenet/pdf/ecoli-shigella-salmonella-pfge-protocol508.pdf). Salmonella serotype Braenderup (strain H9812; American Type Culture Collection catalog no. BAA-664) was used as the marker for all PFGE gels. Briefly, DNA was extracted and digested with XbaI. XbaI PFGE patterns were analyzed for similarity, and a dendrogram was generated by the Bionumerics software, version 6.5 (Applied Maths, Sint Martens-Latem, Belgium) with Dice similarity indices (complete linkage; optimization, 1.5%; position tolerance, 1.5%) and the unweighted-pair group method with arithmetic means. In this study, a cluster was defined as STEC isolates that grouped together in the dendrogram with a Dice similarity index equal or above 80%.

### 5.7. Statistical Analysis

Fisher’s exact test was used to determine if there were statistically significant differences between the proportions of STEC genes (SPSS Statistics 19; IBM, Armonk, NY, USA). A *p*-value <0.05 was considered significant.

## Figures and Tables

**Table 1:** Proportions of essential chromosomal and plasmid-encoded virulence-associated genes in Shiga toxin-producing *Escherichia coli* (STEC) isolates from humans in South Africa.

No	Year	Serotype	*stx1*	*stx2*	*stx2c*	*stx2d*	*eaeA*	*ehxA*	*katP*	*espP*	*etpD*	*saa*	*subA*
**1**	2007	O26:H11	+	−	−	−	+	+	+	+	−	−	−
**2**	2008	O26:H11	+	−	−	−	+	+	+	+	−	−	−
**3**	2010	O111:H8	+	−	−	−	+	+	+	−	−	−	−
**4**	2010	O26:H11	+	−	−	−	+	−	−	−	−	−	−
**5**	2011	O26:H11	+	−	−	−	+	+	+	+	−	−	−
**6**	2011	O157:H7	−	+	+	+	+	+	+	+	+	−	−
**7**	2010	O26:H11	+	−	−	−	+	+	+	+	−	−	−
**8**	2010	O117:H7	+	−	−	−	−	−	−	−	−	−	−
**9**	2010	O157:H7	−	+	+	+	+	+	+	+	+	−	−
**10**	2003	ONT:H8	+	−	−	−	+	−	−	−	−	−	−
**11**	2006	O111:H8	+	−	−	−	+	+	−	−	−	−	−
**12**	2006	O117:H7	+	−	−	−	−	−	−	−	−	−	−
**13**	2006	O117:H7	+	−	−	−	−	−	−	−	−	−	−
**14**	2007	O22:H16	−	+	−	−	−	−	−	−	−	−	−
**15**	2007	O8:H19	+	+	+	+	−	+	−	+	−	+	−
**16**	2007	O107:H7	+	+	−	+	−	+	−	+	−	+	−
**17**	2007	O107:H7	+	+	−	+	−	+	−	+	−	+	−
**18**	2007	O107:H7	+	+	−	+	−	+	−	+	−	+	−
**19**	2007	O113:H21	−	+	+	+	−	+	−	+	−	+	+
**20**	2009	ONT:H21	+	−	−	−	−	−	+	−	−	+	−
**21**	2009	O111:H8	+	−	+	−	−	−	−	−	−	−	−
**22**	2009	O111:H8	+	−	−	−	+	+	−	−	−	−	−
**23**	2009	O156:H7	+	−	−	−	−	−	−	−	−	−	−
**24**	2009	O26:H11	+	−	−	−	+	+	+	+	−	−	−
**25**	2009	O157:H7	+	+	+	+	+	+	+	+	+	−	−
**26**	2009	O111:H8	+	−	−	−	+	+	−	−	−	−	−
**27**	2012	O117:H7	+	−	−	−	−	−	−	−	−	−	−
**28**	2012	O117:H7	+	−	−	−	−	−	−	−	−	−	−
**29**	2012	O157:H7	−	+	+	+	+	+	+	+	+	−	−
**30**	2012	O157:H7	−	+	+	+	+	+	+	+	+	−	−
**31**	2013	O118:H16	+	−	−	−	+	+	+	+	−	−	−
**32**	2013	O156:H7	+	−	−	−	−	−	−	−	−	−	−
**33**	2009	O26:H11	+	−	−	−	+	+	+	+	−	−	−
**34**	2013	O26:H11	+	−	−	−	+	+	+	+	−	−	−
**35**	2008	O111:H8	+	−	−	−	+	+	−	−	−	−	−
**36**	2008	O26:H11	+	−	−	−	+	+	+	+	−	−	−
**37**	2010	O5:HNT	+	−	+	−	+	+	−	+	−	−	−
**38**	2008	O5:HNT	+	−	+	−	+	+	−	+	+	−	−
		TOTAL	32	11	10	10	23	26	16	21	6	6	1
		% Positive	84	29	26	26	61	68	42	55	16	16	3
